# An Interpretable Two-Phase Modeling Approach for Lung Cancer Survivability Prediction

**DOI:** 10.3390/s22186783

**Published:** 2022-09-08

**Authors:** Zahra Sedighi-Maman, Jonathan J. Heath

**Affiliations:** 1 Robert B. Willumstad School of Business, Adelphi University, Garden City, NY 11530, USA; 2 McDonough School of Business, Georgetown University, Washington, DC 20057, USA

**Keywords:** lung cancer, survival prediction, unbalanced data, one-hot encoding, Surveillance, Epidemiology, and End Results (SEER)

## Abstract

Although lung cancer survival status and survival length predictions have primarily been studied individually, a scheme that leverages both fields in an interpretable way for physicians remains elusive. We propose a two-phase data analytic framework that is capable of classifying survival status for 0.5-, 1-, 1.5-, 2-, 2.5-, and 3-year time-points (phase I) and predicting the number of survival months within 3 years (phase II) using recent Surveillance, Epidemiology, and End Results data from 2010 to 2017. In this study, we employ three analytical models (general linear model, extreme gradient boosting, and artificial neural networks), five data balancing techniques (synthetic minority oversampling technique (SMOTE), relocating safe level SMOTE, borderline SMOTE, adaptive synthetic sampling, and majority weighted minority oversampling technique), two feature selection methods (least absolute shrinkage and selection operator (LASSO) and random forest), and the one-hot encoding approach. By implementing a comprehensive data preparation phase, we demonstrate that a computationally efficient and interpretable method such as GLM performs comparably to more complex models. Moreover, we quantify the effects of individual features in phase I and II by exploiting GLM coefficients. To the best of our knowledge, this study is the first to (a) implement a comprehensive data processing approach to develop performant, computationally efficient, and interpretable methods in comparison to black-box models, (b) visualize top factors impacting survival odds by utilizing the change in odds ratio, and (c) comprehensively explore short-term lung cancer survival using a two-phase approach.

## 1. Introduction

Lung cancer is the leading cause of cancer-related deaths worldwide [1]. According to the World Health Organization (WHO) [1], there were 2.9 million new cases and 1.76 million deaths due to lung cancer globally in 2018. It is estimated by the American Cancer Society [2] that around 236,740 people (117,910 men and 118,830 women) will be diagnosed with lung cancer while approximately 130,180 deaths (68,820 men and 61,360 women) will arise in 2022. According to Lemjabbar-Alaoui et al. [3], the prognosis of lung cancer is generally poor despite all the advancements in diagnostics and therapeutics. Through the use of data mining methods, it is possible to further analyze cancer patient data and predict the survivability outcomes. The combination of machine learning methods with physician expertise can help facilitate cancer treatment options. The Surveillance, Epidemiology, and End Results (SEER) [4] program is currently the most comprehensive repository that contains clinical data for approximately 34.6% of the US population with cancer. We believe that the literature on lung cancer survivability using SEER data can be classified within two main research groups. The first group [5,6,7,8,9,10,11,12] focuses on using statistical methods (e.g., Cox regression, Kaplan–Meier methods, and chi-squared test) for survival analysis as well as finding significant prognostic features (e.g., tumor size, performing surgery, and positive lymph node ratio) that influence survival. The second group focuses on using machine learning methods for survival prediction (see Table 1). In this study, we focus predominantly on the second group of research, while identifying significant prognostic features.

Survival status prediction, length of survival estimation, and cancer patient clustering are primary topics found in the machine learning literature that utilizes the SEER dataset, where focus is placed on model accuracy. Moreover, common classification, clustering, and regression models employed within the second group of research include artificial neural networks (ANNs), support vector machines (SVMs), Naïve Bayes (NB), decision trees (DTs), random forest (RF), ensemble methods, K-means, and bidirectional data partitioning (BDP) [7,13,14,15,16,17,18,19,20,21,22]. Apart from the great strides made in lung cancer prediction research, several challenges still exist:

- Although most studies explore survival status classification [7,14,15,17,18,21,22] and survival length prediction [19] individually, a scheme that leverages both remains elusive [13,16].
- Data used in lung cancer survivability predictions suffer from the class imbalance problem, which produces algorithm bias in favor of the majority class. This issue is scarcely addressed in cancer-related studies [14].
- Most features in the SEER data are categorical (e.g., grade, stage, and race). Many studies adopt integer encoding [14,15,16,21,22] to transform categorical features, which can introduce improper hierarchical order in feature levels. Alternatively, several studies [7,13,17,22] apply one-hot encoding to remedy non-ordinal relationships; however, most of these studies omit feature interpretation in favor of model performance.
- An interpretable yet effective model for predicting lung cancer survivability or survival length, which can assist a practitioner in their decision-making process, remains missing.

This paper lays out a two-phase data analytic framework, where phase I predicts the 6-month (0.5-year), 1-, 1.5-, 2-, 2.5-, and 3-year survival status of patients diagnosed with lung cancer while phase II predicts the number of survival months for patients who succumb to lung cancer within 3 years. In phase I, we use three analytical models (general linear model (GLM), extreme gradient boosting (XGB), and ANN) along with five data balancing techniques (synthetic minority oversampling technique (SMOTE), relocating safe level SMOTE (RSLSMOTE), borderline SMOTE (BLSMOTE), adaptive synthetic sampling (ADASYN), and majority weighted minority oversampling technique (MWMOTE)), and two feature selection methods (least absolute shrinkage and selection operator (LASSO) and RF), while using the one-hot encoding approach to encode the categorical features. In phase II, we employ similar models used in phase I (GLM, XGB, and ANN) along with two feature selection methods (LASSO and RF) to predict the number of survival months for deceased patients within 3 years. We extract and interpret significant predictors based on regression coefficients for phase I (using odds ratio) and phase II. Through our proposed data analytic framework, we address the four challenges mentioned above. Furthermore, by implementing a comprehensive data preparation phase, we demonstrate that a statistical approach such as GLM performs comparably to the more complex models (e.g., XGB and ANN) at a considerably lower computational cost. The remaining parts of the paper are organized as follows: our proposed data analytic framework is discussed in detail within Section 2, results are presented and discussed in Section 3, and concluding remarks, including research limitations and future outlook, are given in Section 4.

## 2. Material and Methods

**Figure 1:**
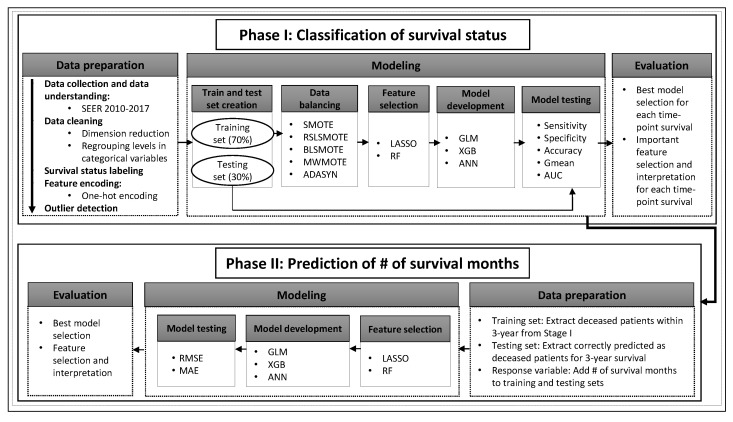
A proposed two-phase data analytic framework for lung cancer survivability prediction.

Figure 1 presents a diagram illustrating our proposed two-phase data analytic framework for lung cancer survivability prediction, where each phase encompasses several steps. In the following subsections, various phases are described in detail.

### 2.1. Phase I: Classification of Survival Status

#### 2.1.1. Data Preparation

The data preparation phase comprises five main steps: (1) data collection and data understanding, (2) data cleaning, (3) survival status labeling, (4) feature encoding, and (5) outlier detection, which are discussed in the following paragraphs.

This study incorporates de-identified diagnosed lung cancer records from the SEER dataset (November 2020 Submission) spanning from January 2010 to December 2017, where additional features were introduced to the SEER database in 2010 while several of these features were later omitted in 2018. As shown in Table 2, various criteria/filters are applied during data collection, resulting in 183 features and 129,756 records.

Due to a large number of unknown/missing values, duplicate variables, and correlated features, the filtered lung cancer dataset requires extensive data cleansing. In this step, features are identified and removed when (a) variables are discontinued or lack longitudinality, (b) variables possess more than 80% missing values, (c) variables are repetitive, and (d) variables contain constant input. Rather than arbitrarily removing records that contain NA values, the unknown and NA levels in categorical features are combined, which reduces data dimensionality while preserving statistical power. Similar to a past study [23], the categorical levels with frequencies less than 5% are regrouped in order to avoid overfitting and to avoid introducing bias to a model. Additionally, in order to mitigate gratuitous model bias from imputation, records with unknown values for *total number of the benign tumors* and *regional node examined* are removed, maintaining feature distributions. After the data cleaning step, 22 features and 125,498 records remain. All features and their type are listed in Table 3, where 4 features are numerical and 18 are categorical.

In the third step of the data preparation phase, the survival status (response variable) is generated for each patient at each time point using the *survival months* feature. For example, if *survival months* < 6, then the survival status will be denoted as 0, which indicates death within 6 months. Otherwise, the survival status is assigned as 1, which indicates that the patient survived for 6 months or greater.

In the fourth step, categorical features are transformed to ensure that the dataset is prepared in a format applicable to analytical models. As shown in Table 3, most features are categorical; however, machine learning models rely on numerical features for input. Many researchers [14,15,16,21,22] employ integer encoding to re-code categorical features, where the levels in each categorical feature are assigned integer values (e.g., denoting Grade I, Grade II, and Grade III as 1, 2, and 3). Instead, we opt for the one-hot encoding approach, which circumvents improper hierarchical order and encodes a categorical feature with *m* levels into m−1 dummy variables, to avoid multicollinearity [24]. After feature encoding, we ended up with 60 features in our dataset.

In the fifth and final step, we utilize Cook’s distance [25] to eliminate outlier incidences in our dataset. Cook’s distance is one of the most popular approaches for detecting outliers [26], and it offered modest refinements in our preliminary analyses. For each observation, the Cook’s distance is determined by comparing the fitted model performance with and without the data point. Observations with high Cook’s distances are considered influential or outliers. We adopt a threshold of 4/n for outlier detection, a standard threshold in the literature [27].

#### 2.1.2. Modeling

In this phase, the dataset is randomly split into training (70%) and testing (30%) sets. In addition to using 5-fold cross-validation, bootstrapping is utilized during model training to mitigate overfitting and reduce model variance. Due to a disproportionate number of survival and deceased class instances existing for each time-point, class distributions within the training set are adjusted to address the class imbalance problem. Based on the superiority of synthetic sampling demonstrated in previous studies [28,29], we explore five re-sampling approaches: SMOTE [30], RSLSMOTE [31], BLSMOTE [32], ADASYN [33], and MWMOTE [34].

It is important to note that the one-hot encoding approach increases the number of features, which increases the complexity of model development and the training process. Traditional feature selection methods (such as forward/backward selection and recursive feature elimination) are not practical for high-dimensional data. Therefore, two popular embedded feature selection methods, namely, LASSO [35] and RF, are used to reduce the dimension of the input features. Both of these methods are widely used in the literature to extract important features from high-denominational data [36,37]. Feature selection is used to decrease the complexity while increasing the generalizability of the analytical model.

Next, three popular models (GLM, XGB, and ANN) from three analytical groups, (a) statistical models, (b) ensemble models, and (c) deep learning models, are used for model development. Statistical models are simple, computationally efficient, and more interpretable compared to ensemble and deep learning models. Ensemble models typically offer high prediction performance by leveraging a majority voting approach, where the results of many lesser classifiers are combined. ANN models also offer high prediction performance through variable transformations; however, their required computational time notably increases as the dimensionality of a dataset increases [38]. These three models, drawn from three common analytical groups, are carefully selected in order to gauge how prediction performance varies from a simpler (GLM) to a more complex analytical model (ANN). In terms of complexity, the three models can be categorized as less complex (GLM), mid-complex (XGB), and complex (ANN). Furthermore, we inquire whether comprehensive data preprocessing can substitute complex models (XGB and ANN) with simpler models (GLM). For further details regarding these data mining methods, we refer the readers to [39,40,41]. To evaluate model prediction performance, we compute five metrics: (a) sensitivity—the reliability of survival status prediction, (b) sensitivity—the reliability of decease status prediction, (c) accuracy—a measure of the overall survival and decease status prediction performance, (d) G-mean—the combined reliability of survival and decease status prediction (pertinent to imbalanced datasets), and (e) area under the receiver operating characteristic (ROC) curve (AUC)—a measure of the diagnostic accuracy for survival and decease status prediction. Note that we use G-mean as our primary criterion for model selection, where a model with a higher G-mean value is more reliable in simultaneously predicting survival and decease statuses. The leading four metrics are listed as follows:

(1) $$Sensitivity=\frac{TP}{TP+FN},$$

(2) $$Specificity=\frac{TN}{TN+FP},$$

(3) $$Accuracy=\frac{TP+TN}{TP+TN+FP+FN},$$

(4) $$G-mean=\sqrt{Sensitivity\times Specificity},$$

where TP, TN, FP, and FN refer to the number of true positives, true negatives, false positives, and false negatives, respectively.

### 2.2. Phase II: Prediction of the Number of Survival Months

The goal of phase II is to predict the number of survival months for patients predicted to die within 3 years. The initial (full featured and unbalanced) 3-year survival dataset utilized in phase I is used to construct the training dataset in phase II, where we include an additional *number of survival month* feature. The testing dataset for phase II is the correctly predicted output from phase I. Moreover, the model development phase is similar to phase I. In addition to using LASSO and RF methods for feature selection, we employ GLM, XGB, and ANN to predict the number of survival months. To gauge the performance of each prediction model, we calculate the root mean squared error (RMSE) and mean absolute error (MAE). These metrics are listed in Equations (5) and (6), where $Y_{i}$ is the actual number of survival months, ${\hat{Y}}_{i}$ is the predicted number of survival months, and *m* is the number of records:

(5) $$RMSE=\sqrt{\frac{1}{m}\sum_{i=1}^{m}{\left(Y_{i}-{\hat{Y}}_{i}\right)}^{2}},$$

(6) $$MAE=\frac{1}{m}\sum_{i=1}^{m}\left|Y_{i}-{\hat{Y}}_{i}\right|.$$

## 3. Results and Discussion

In this section, we present the prediction results for phases I and II. For phase I, due to the computational cost of XGB and ANN models, we prune the total number of model combinations, which take into account two feature selection methods and five resampling approaches. Firstly, we develop all model combinations for 1-year survival prediction and identify the best feature selection method and data balancing technique for GLM, XGB and ANN models (Table 4). These initial model benchmarks are based on 1-year survival data, which contain the largest number of observations compared to other time-points, with the exception of six-month survival. In addition to having a substantial sample size (high reliability), 1-year survival is one of the most commonly reported time-points in the literature [7,15,17]. We use these benchmark results to delimit the best feature selection and data balancing methods. Next, we combine the top feature selection and data balancing techniques found in Table 4 with GLM, XGB, and ANN for 0.5-, 1.5-, 2-, 2.5-, and 3-year survival prediction (Table 5).

### 3.1. Phase I: Classification

Table 4 presents the classification results for 1-year survival prediction. Firstly, LASSO feature selection performs marginally better than RF feature selection across all models and all data balancing techniques using G-mean as a criterion. The G-mean values range between 0.847–0.870 and 0.846–0.858 for all models using LASSO and RF feature selection, respectively. Note that LASSO is computationally efficient compared to RF feature selection. Second, the use of ADASYN for data balancing provides equal or higher G-mean values (0.855–0.870) across all models compared to the remaining four data balancing techniques. Models utilizing balancing techniques such as SMOTE and MWMOTE are among the top performing models just below the ADASYN method. The best-performing GLM, XGB, and ANN models based on the G-mean metric (marked in bold in Table 4) are used in 0.5-, 1.5-, 2-, 2.5-, and 3-year survival predictions.

**Figure 2:**
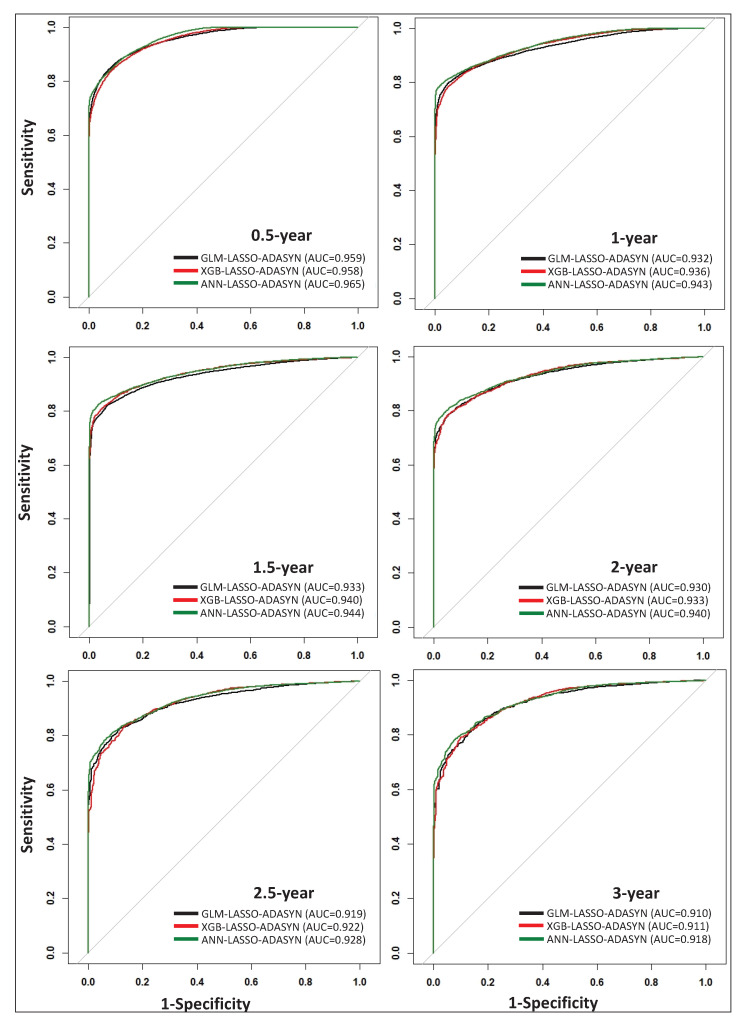
Phase I ROC curves for 0.5-, 1-, 1.5-, 2-, 2.5-, and 3-year survival time-points (18 models).

Table 5 presents the classification results for 0.5-, 1-, 1.5-, 2-, 2.5-, and 3-year survival predictions using GLM, XGB, and ANN, along with LASSO feature selection and the ADASYN data balancing technique. The highest-performing models for each of the six time-points are marked in bold using the G-mean value as a criterion. Based on Table 5, GLM is the top model for 0.5-year survival prediction, with a G-mean value of 0.887, while ANN is the top-performing model for 1-, 1.5-, 2-, 2.5-, and 3-year survival prediction. Although ANN models exhibit higher performance compared to GLM and XGB for 1-, 1.5-, 2-, 2.5-, and 3-year survival prediction, the G-mean values for GLM and XGB are nearly on par with those offered by ANN models. Additionally, ROC curves for all models listed in Table 5 are plotted in Figure 2, which visually demonstrates the comparable performance of each technique. By incorporating a thorough data scheme within our model framework, we demonstrate that simple models such as GLM can perform comparably to more complex models such as XGB and ANN.

### 3.2. Important Features for Survival Prediction

We use the GLM–LASSO–ADASYN models to extract the topmost significant survival predictors for all time-points (see supplementary materials https://github.com/zahrame/LungCancerPrediction for a list of GLM equations). Besides their interpretability, GLM models provide relatively high classification results (see Table 5) at low computational cost. We define the odds ratio (OR $=\frac{p}{1-p}$ in which *p* is the probability of survival) and calculate the relative change in OR (ΔOR) to quantify the impact of each important feature based on its respective GLM coefficient:

(7) $$log\left(\frac{p}{1-p}\right)=\beta_{0}+\beta_{1}x_{1}+\beta_{2}x_{2}+\beta_{3}x_{3}+...+\beta_{i}x_{i},\;i=\;\#\;\mathrm{of}\;\mathrm{the}\;\mathrm{feature}\;\mathrm{in}\;\mathrm{the}\;\mathrm{model}$$

(8) $$\Delta \mathrm{OR}{}_{j}=\frac{{\mathrm{OR}}_{new}-{\mathrm{OR}}_{old}}{{\mathrm{OR}}_{old}}=\exp\beta_{j}-1,\;j=\;\mathrm{feature}\;j\;\mathrm{in}\;\mathrm{the}\;\mathrm{model}.$$

**Figure 3:**
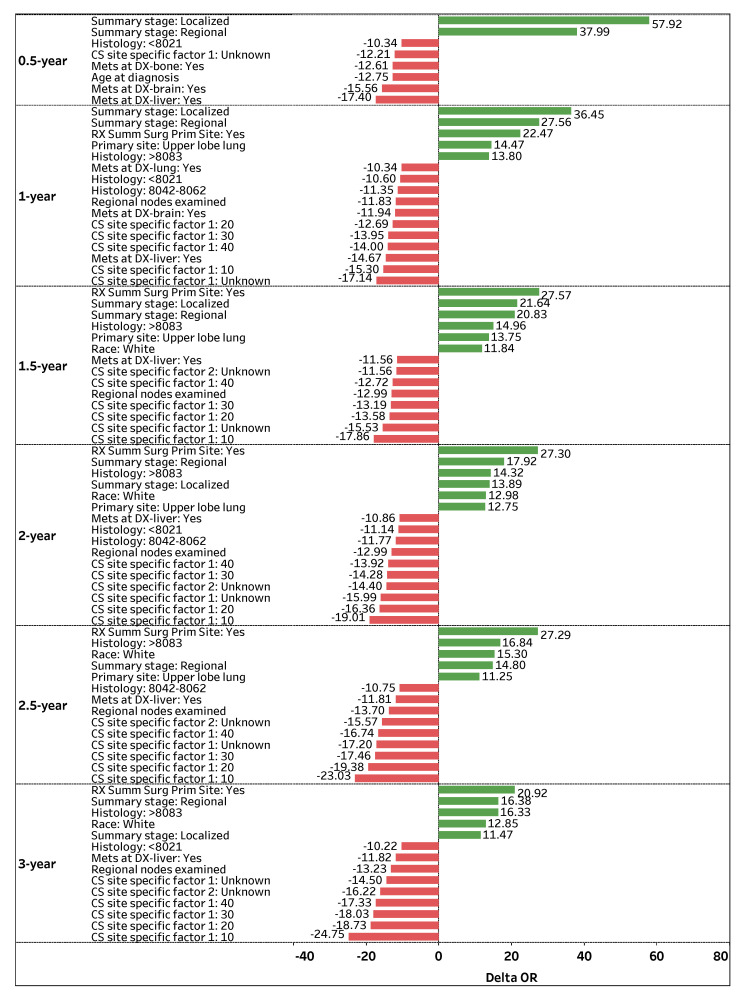
Top (phase I) predictors with ΔOR >|10%| for 0.5-, 1-, 1.5-, 2-, 2.5-, and 3-year survival time-points.

By defining the difference between the odds (${\mathrm{OR}}_{new}-{\mathrm{OR}}_{old}$) of an individual feature increasing by one unit ($x_{j}+1$) and exponentiating both sides of the equation, we can decouple each feature’s effect on the odds of survival (confined within the logarithmic function of Equation (7)). By subtracting one from the results, we obtain the effective change in the odds ratio (Equation (8)) by an individual feature [23]. Figure 3 visualizes the top-contributing features with ΔOR values greater than |10%| for 0.5-, 1-, 1.5-, 2-, 2.5-, and 3-year survival predictions. The green positive (red negative) bars correspond to an increase (decrease) in the odds of survival.

*Summary stage: Regional* is a highly significant and consistent feature that positively impacts (ΔOR > 0) a patient’s odds of survival across all time-points. If the spread of lung cancer (*Summary stage*) in a patient is categorized as *Regional*, the odds of survival are 37.99%, 27.56%, 21.64%, 17.92%, 14.80%, and 16.38% higher on average (holding other features constant) for 0.5-, 1-, 1.5-, 2-, 2.5-, and 3-year survival time-points, respectively. Similarly, *Summary stage: Localized* is a significant feature that positively affects a patient’s survival status, particularly for early time-points. If the spread of lung cancer is categorized as *Localized*, the odds of survival are 57.92%, 36.45%, 21.64%, 13.89%, and 11.47% higher on average (holding other features constant) for 0.5-, 1-, 1.5-, 2-, and 3-year time-points, respectively.

Another prominent feature that positively contributes to patient survival is *RX Summ Surg Prim Site: Yes*, a feature that documents if a surgery procedure is performed on the primary cancer site. Figure 3 shows that if surgery is performed on a primary site, a patient’s odds of survival are 22.47%, 27.57%, 27.30%, 27.29%, and 20.92% higher on average (holding other features constant) for 1-, 1.5-, 2-, 2.5-, and 3-year survival time-points, respectively. Regarding primary cancer sites, *Primary site: Upper lobe lung* is attributed to higher odds of survival for several time-points. If the primary cancer site of a patient is *Upper lobe lung*, the patient’s odds of survival are 14.47%, 13.75%, 12.75%, and 11.25% higher on average (holding other features constant) for 1-, 1.5-, 2-, and 2.5-year survival time-points, respectively.

In contrast, *CS site specific factor 1: Unknown* is one of the most significant and consistent features that negatively impacts (ΔOR < 0) a patient’s odds of survival across all time-points. If the existence of separate tumor nodules (*CS site specific factor 1*) cannot be assessed in a patient’s ipsilateral lung, the odds of survival are 12.21%, 17.14%, 15.53%, 15.99%, 17.20%, and 14.5% lower on average (holding other features constant) for 0.5-, 1-, 1.5-, 2-, 2.5-, and 3-year survival time-points, respectively. Note that the presence of separate tumor nodules in the ipsilateral lung (*CS site specific factor 1: 10, 20, 30,* and *40*) is highly significant, which negatively impacts (ΔOR < 0) a patient’s survival status for 1-, 1.5-, 2-, 2.5-, and 3-year survival time-points.

*Mets at DX-liver: Yes* is another significant and consistent feature that negatively affects a patient’s odds of survival. If a patient experiences a distant metastatic involvement of the liver, the odds of survival are 17.40%, 14.67%, 11.56%, 10.86%, 11.81%, and 11.82% lower on average (holding other features constant) for 0.5-, 1-, 1.5-, 2-, 2.5-, and 3-year survival time-points, respectively. Moreover, *Regional nodes examined* is a vital feature that negatively affects a patient’s odds of survival. If the number of removed and examined regional lymph nodes for a patient increases by one node, the patient’s odds of survival are 11.83%, 12.99%, 12.99%, 13.7%, and 13.23% lower (holding other features constant) for 1-, 1.5-, 2-, 2.5-, and 3-year survival time-points, respectively.

### 3.3. Phase II: Regression

Table 6 presents the number of survival months prediction results for deceased patients within 3 years, where the best models are marked in bold. Similar to phase I, LASSO outperforms RF feature selection with marginally smaller values of RMSE and MAE for each model methodology. The GLM and XGB models offer similar survival month prediction performance with an MAE ∼ 5.6 months. Even though ANN is a more complex model compared to GLM and XGB, the MAE values for ANN using LASSO and RF feature selection are ∼6.7 and ∼7.1 months, respectively. These findings illustrate that although ANN outperforms GLM and XGB in classification problems (phase I), ANN is not guaranteed to outperform the simpler models in regression problems (phase II).

**Figure 4:**
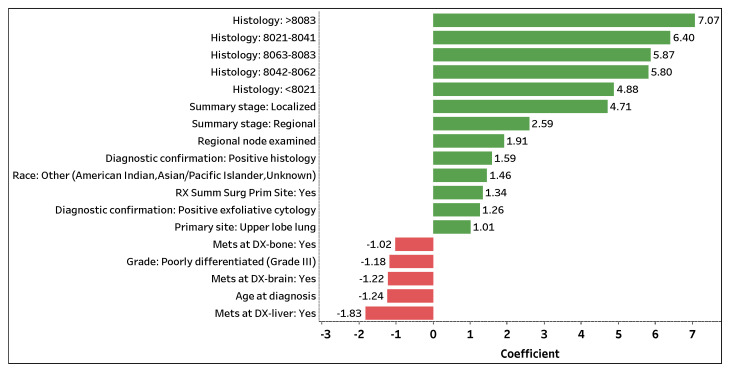
Top (phase II) predictors with coefficient values >|1.00| that predict the number of survival months.

Similar to phase I, we use the GLM–LASSO model to extract significant features and their coefficients (see supplementary https://github.com/zahrame/LungCancerPrediction for a list of GLM equations). Figure 4 visualizes the top 18 contributing features with coefficient values greater than |1.00| that predict the number of survival months. The 13 (5) features with positive green (negative red) bars are attributed to an increase (decrease) in the number of survival months. *Histology*: >8083 is the topmost significant feature that positively impacts the number of survival months. If a patient (predicted to perish) is assigned a histology code greater than 8083, the patient is expected to survive 7.07 months longer on average (holding other features constant). Note that a patient (predicted to perish) assigned a histology code, regardless of carcinoma group type, is expected to live several months longer on average compared to a patient who was not or could not be assigned a code (holding other features constant).

*Summary stage: Localized* and *Summary stage: Regional* are the next important features that positively contribute to the number of survival months. If the spread of lung cancer in a patient (predicted to perish) is localized or regional, the patient is expected to survive 4.71 or 2.59 months longer on average (holding other features constant), respectively. Additionally, *Regional nodes examined* and *RX Summ Surg Prime Site: Yes* are significant features in predicting the number of survival months of a lung cancer patient. If a patient (predicted to perish) has an additional lymph node removed and examined or has surgery performed on a primary cancer site, the patient is expected to live 1.91 or 1.34 months longer on average (holding other features constant). Note that a higher number of examined regional lymph nodes implies a decrease in a patient’s odds of survival (phase I); yet, with the removal and examination of additional lymph nodes, the survival length of a patient expected to perish may be prolonged (holding other features constant).

Contrarily, *Mets at DX-liver: Yes* is the top significant feature that negatively affects the number of survival months. If distant liver metastases have formed in a patient (predicted to perish), the patient is expected to live 1.83 months less on average (holding other features constant). Moreover, if distant brain (*Mets at DX-brain: Yes*) or bone (*Mets at DX-bone: Yes*) metastases have formed in a patient (predicted to perish), the patient is expected to live 1.22 or 1.02 months less on average (holding other features constant), respectively. For every additional year in age (*Age at diagnosis*), a patient (predicted to perish) is expected to live 1.24 months less on average (holding other features constant). Lastly, if a patient (predicted to perish) is diagnosed with Grade III lung cancer (*Grade: Poorly differentiated (Grade III)*), the patient is expected to live 1.18 months less on average (holding other features constant). Similar to phase I, the use of one-hot encoding enables us to not only extract significant categorical levels but to interpret the individual levels.

### 3.4. Recent Literature Comparison

In spite of the fact that a proper one-to-one comparison between our research and prior lung cancer data mining studies is not possible due to variations in dataset time ranges, feature availability, data collection criteria, data preprocessing techniques, modeling approaches, and prediction time-points, we highlight some similarities and differences to provide a synopsis. In a recent study, Doppalapudi et al. [13] yielded AUC values as high as 0.83, 0.86, and 0.92 for 0.5-, 0.5–2-, and >2-year survival prediction, respectively, based on 2004–2016 SEER data using CNN. Our data and approach yield AUC values as high as 0.97, 0.94, 0.94, 0.94, 0.93, and 0.92 for 0.5-, 1-, 1.5-, 2-, 2.5-, and 3-year time-points, respectively (Figure 2). Similar to our study, Doppalapudi et al. found that *Histology*, *Age at diagnosis*, *Summary stage*, and *Primary site* are important lung cancer survival predictors. Unlike our results, Doppalapudi et al. found that *Registry information*, *Sex*, *Number of radiation rounds*, and two discontinued variables (*Number of lymph nodes* and *Derived AJCC TNM*) in the SEER dataset are important features. Although this study reports the relative importance of various contributing features in survival prediction, the effect of each feature is not quantified.

In another recent study, Wang et al. [7] achieved accuracies (AUC was not reported) of 0.93, 0.78, and 0.72 for 1-, 3-, and 5-year survival prediction, respectively, based on 2010–2015 SEER data using XGB and LR. Our study yields accuracies as high as 0.89, 0.86, 0.87, 0.86, 0.85, and 0.84 for 0.5-, 1-, 1.5-, 2-, 2.5-, and 3-year time-points, respectively. The important predictors *Surgery*, *Grade*, *Histology*, *Age at diagnosis*, and *Race* found by Wang et al. are consistent with our results; however, *Laterality*, *Sex*, *Marital status*, and *Derived AJCC TNM* (a discontinued variable in SEER data) are not. In addition, Jonson et al. [14] yielded an AUC value of 0.94 for 5-year survival prediction based on 1975–2015 SEER data using RF and AdaBoost models. Although Jonson et al. explored intermediate-term survival, they found that *Age at diagnosis*, *Histology*, *Surgery on primary site*, and *Summary stage* are important features for survival prediction, similarly found in our study for short-term survival. Jonson et al. also found that *Sequence Number* (used as one of our criteria for data collection) and two discontinued variables (*Number of lymph nodes* and *Extent of disease*) are important predictors, which differ from our study. Again, the impact of each feature on lung cancer survival is not quantified in the latter two studies. 

## 4. Conclusions

Pertaining to the results obtained in this study, we have three main contributions, previously unexplored in lung cancer data mining research. First, we developed a two-phase data analytic framework that is capable of 1) predicting the survival status of a patient with lung cancer for 0.5-, 1-, 1.5-, 2-, 2.5-, and 3-year time-points and 2) predicting the number of survival months for patients who were predicted and labeled as deceased within 3 years. Second, by incorporating a comprehensive data preprocessing step, we showed that a computationally efficient and interpretable model such as GLM can perform comparably to complex models such as XGB and ANN. Moreover, the data preparation steps outlined in phases I and II facilitate data reproducibility. Third, we used GLM–LASSO–ADASYN models to extract important numerical and encoded categorical features (using one-hot encoding), where we interpreted the effect of individual features on the odds of survival in phase I. Similarly, in phase II, we used the GLM–LASSO model to extract important numerical and individual categorical features (using one-hot encoding) that influence the number of predicted survival months. Although the performance of the proposed framework in practice is still a challenge, since other potential factors such as a patient’s lifestyle (e.g., diet and smoking behavior) or prior medical/drug history may impact lung cancer survivability, our simple yet interpretable GLM models (phases I and II) may assist physicians in better decision-making by prioritizing the most important factors related to lung cancer survivability.

## Figures and Tables

**Table 1:** A summary of recent statistical and data mining research in lung cancer using SEER data.

Category	Paper	Data, # of Variables, Objective	Method/Model
**Statistical**	[5]	SEER 2010–2016, 23 variables, univariate and multivariate analysis	Cox R
	[6]	SEER 2008–2016, 13 variables, univariate and multivariate analysis	Cox R, Kaplan–Meier
	[7]	SEER 2010–2015, 12 variables, univariate and multivariate analysis	Cox R, Kaplan–Meier, chi-squared test
	[8]	SEER 2001–2014, 12 variables, univariate and multivariate analysis	Cox R, Kaplan–Meier, chi-squared test
	[9]	SEER 2010–2013, 12 variables, multivariate analysis	Cox R, Kaplan–Meier, chi-squared test
	[10]	SEER 2006–2010, # of variables is not disclosed, univariate and multivariate analysis	Cox R, Kaplan–Meier, chi-squared test, ANOVA
	[11]	SEER 1998–2009, 13 variables, univariate and multivariate analysis	Kaplan–Meier, chi-squared test
	[12]	SEER 1988–2006, # of variables is not disclosed, univariate and multivariate analysis	Cox R, Kaplan–Meier
**Data Mining**	[13]	SEER 2004–2016, # of variables is not disclosed, classification + regression for 3 categories: ≤6 months, 7–24 months, and ≥24 months	ANN, RNN, CNN, RF, SVM, NB, GBM, LR
	[7]	SEER 2010–2015, 12 variables, classification (1-, 3-, 5-year survival)	XGB, LR, NB, DT, KNN, RF, SVM
	[14]	SEER 2010–2015, 14 variables, classification (5-year survival)	LR, NB, Gaussian K-base NB,
	[15]	SEER 1973–2012, 114 variables, classification (0.5-, 1-, 5-year survival)	RF, ANN
	[16]	SEER 2004–2009, 13 variables, classification + regression for 3 categories: ≤6 months, 7–24 months, and ≥24 months	GBM, RF, GLM, EV
	[17]	SEER 2004–2009, 48 variables, classification (1-, 5-year survival)	LR, MLP
	[18]	SEER, 24 variables, classification (5-year survival)	RF, BA, DA, ADB, BOO, RS, RIPPER, DS, Simple Cart, DT, SMO, LR, BN
	[19]	SEER 2004–2009, 8 variables, clustering + regression	MBC, K-Means, GBM, SOM, HC, NNMF, PCA, LR
	[20]	SEER 1998–2008, 45 variables, classification (0.67-year survival) + clustering	BDP, K-Means, KNN, J48
	[21]	SEER 1998–2006, 64 variables, classification (0.5-, 0.75-, 1-, 2-, 5-year survival)	DT, RF, LB, RS, ADT, EV, ANN, SVM, DS
	[22]	SEER 1988–2004, 13 variables, classification (5-, 7-, 10-year survival)	NB, DT

Cox R = Cox regression, RNN = recurrent neural network, CNN = convolutional neural network, GBM = gradient boosting machine, LR = logistic regression, XGB = extreme gradient boosting, KNN = K-nearest neighbor, GLM = generalized linear model, EV = ensemble voting, MLP = multilayer perceptron, BA = bagging, DA = dagging, ADB = AdaBoost, BOO = boosting, RS = random subspace, DS = decision stump, SMO = sequential minimal optimization, BN = Bayes Net, MBC = model-based clustering, SOM = self-ordering map, HC = hierarchical clustering, NNMF = non-negative matrix factorization, PCA = principal component analysis, CE = custom ensemble, LB = LogitBoost, ADT = alternating DT.

**Table 2:** The criteria/filters applied for data collection.

#	Feature	Criteria/Filters
1	Survival months	= “Complete dates are available and there are more than 0 days of survival”
2	Age at diagnosis	≠ “Unknown”
3	Site record ICD-0-3/WHO 2008	=“Lung and Bronchus”
4	AYA site record/WHO 2008	=“8.3 Carcinoma of trachea, bronchus, and lung”
5	ICCC site rec extended ICD-O-3/WHO 2008	=“XI(f.4) Carcinomas of lung”
6	SEER cause-specific death classification	=“Death attribute to this cancer dx”
7	COD to site record	=“Lung and Bronchus”
8	COD to site rec KM	=“Lung and Bronchus”
9	Behavior recode for analysis	=“Malignant”
10	Behavior code ICD-0-3	=“Malignant”
11	SequenceNumber	=“One Primary Only”

**Table 3:** The resulting features after data preparation with brief descriptions provided.

#	Feature	Description	Type
1	Survival months	# of months that a patient survived after diagnosis	Numerical
2	Age at diagnosis	Age of the patient at diagnosis	Numerical
3	Total number of the benign tumors	# of the benign tumors at diagnosis	Numerical
4	Regional nodes examined	# of the regional lymph nodes examined and removed	Numerical
5	Race	Patient’s race	Categorical
6	Sex	Patient’s sex	Categorical
7	Primary site	Original location of the tumor	Categorical
8	Histology	Composition of cancer tissues	Categorical
9	Grade	Appearance of the tumor and its differentiation	Categorical
10	Laterality	Side of the body that tumor presents	Categorical
11	Diagnostic confirmation	Method(s) used in order to affirm the existence of the tumor	Categorical
12	Summary stage	Extent of disease (EOD) (e.g., regional, distant)	Categorical
13	RX Summ–Surg Prim Site	Surgical procedure performed on the primary site as first course of therapy	Categorical
14	RX Summ–Scope Reg LN Sur	Procedure for removal/biopsy/aspiration of the lymph nodes	Categorical
15	RX Summ–Surg Oth Reg/Dis	Surgical procedure performed beyond the regional lymph nodes	Categorical
16	Reason no cancer-directed surgery	Reason(s) for not performing surgical procedure	Categorical
17	Mets at DX-bone (added in 2015)	Presence of distant metastatic involvement of bone during diagnosis	Categorical
18	Mets at DX-brain (added in 2015)	Presence of metastatic brain disease during diagnosis	Categorical
19	Mets at DX-liver (added in 2015)	Presence of distant metastatic involvement of the liver	Categorical
20	Mets at DX-lung (added in 2015)	Presence of distant metastatic involvement of the lung	Categorical
21	CS Site-Specific Factor 1	Additional information to identify cancer stage	Categorical
22	CS Site-Specific Factor 2	Additional information to identify cancer stage	Categorical

**Table 4:** Mean performance (and corresponding standard deviation) of phase I classifiers for 1-year survival. Top models are marked in **bold** for convenience.

Model	Sensitivity	Specificity	Accuracy	G-Mean	AUC
GLM–LASSO–SMOTE	0.863 (0.001)	0.855 (0.002)	0.862 (0.001)	0.859 (0.001)	0.937 (0.000)
GLM–LASSO–RSLSMOTE	0.868 (0.001)	0.839 (0.001)	0.865 (0.000)	0.853 (0.001)	0.937 (0.000)
GLM–LASSO–BLSMOTE	0.843 (0.001)	0.883 (0.002)	0.848 (0.001)	0.863 (0.001)	0.931 (0.000)
GLM–LASSO–MWMOTE	0.859 (0.001)	0.858 (0.002)	0.858 (0.001)	0.858 (0.000)	0.935 (0.000)
**GLM–LASSO–ADASYN **	**0.840 (0.001)**	**0.890 (0.002)**	**0.846 (0.000) **	**0.864 (0.001)**	**0.932 (0.000)**
GLM–RF–SMOTE	0.859 (0.001)	0.839 (0.002)	0.856 (0.001)	0.849 (0.001)	0.933 (0.000)
GLM–RF–RSLSMOTE	0.869 (0.001)	0.824 (0.001)	0.864 (0.000)	0.846 (0.001)	0.932 (0.000)
GLM–RF–BLSMOTE	0.843 (0.001)	0.853 (0.003)	0.844 (0.000)	0.848 (0.001)	0.922 (0.000)
GLM–RF–MWMOTE	0.850 (0.001)	0.859 (0.002)	0.852 (0.001)	0.855 (0.001)	0.932 (0.000)
GLM–RF–ADASYN	0.843 (0.001)	0.867 (0.002)	0.846 (0.001)	0.855 (0.001)	0.929 (0.000)
XGB–LASSO–SMOTE	0.855 (0.003)	0.858 (0.006)	0.855 (0.002)	0.857 (0.002)	0.937 (0.000)
XGB–LASSO–RSLSMOTE	0.865 (0.002)	0.830 (0.005)	0.861 (0.001)	0.847 (0.002)	0.935 (0.000)
XGB–LASSO–BLSMOTE	0.850 (0.001)	0.859 (0.004)	0.851 (0.001)	0.855 (0.001)	0.933 (0.001)
XGB–LASSO–MWMOTE	0.852 (0.002)	0.859 (0.004)	0.853 (0.001)	0.855 (0.002)	0.935 (0.000)
**XGB–LASSO-ADASYN**	**0.850 (0.002)**	**0.867 (0.004)**	**0.852 (0.001)**	**0.859 (0.002)**	**0.936 (0.000)**
XGB–RF–SMOTE	0.857 (0.002)	0.850 (0.005)	0.856 (0.002)	0.853 (0.002)	0.936 (0.000)
XGB–RF–RSLSMOTE	0.868 (0.002)	0.830 (0.003)	0.863 (0.002)	0.849 (0.001)	0.934 (0.000)
XGB–RF–BLSMOTE	0.848 (0.002)	0.848 (0.004)	0.848 (0.002)	0.848 (0.001)	0.929 (0.001)
XGB–RF–MWMOTE	0.851 (0.002)	0.846 (0.005)	0.850 (0.001)	0.848 (0.002)	0.932 (0.000)
XGB–RF–ADASYN	0.850 (0.001)	0.861 (0.004)	0.851 (0.001)	0.855 (0.002)	0.935 (0.001)
ANN–LASSO–SMOTE	0.858 (0.007)	0.872 (0.016)	0.860 (0.005)	0.865 (0.004)	0.945 (0.001)
ANN–LASSO–RSLSMOTE	0.862 (0.007)	0.867 (0.011)	0.863 (0.005)	0.864 (0.003)	0.945 (0.001)
ANN–LASSO–BLSMOTE	0.857 (0.005)	0.878 (0.010)	0.859 (0.003)	0.867 (0.003)	0.942 (0.002)
ANN–LASSO–MWMOTE	0.855 (0.004)	0.881 (0.011)	0.858 (0.002)	0.868 (0.004)	0.942 (0.002)
**ANN–LASSO–ADASYN**	**0.851 (0.004)**	**0.888 (0.005)**	**0.856 (0.003)**	**0.870 (0.002)**	**0.943 (0.001)**
ANN–RF–SMOTE	0.861 (0.004)	0.851 (0.010)	0.860 (0.003)	0.856 (0.003)	0.942 (0.001)
ANN–RF–RSLSMOTE	0.866 (0.006)	0.843 (0.011)	0.863 (0.005)	0.854 (0.003)	0.942 (0.001)
ANN–RF–BLSMOTE	0.856 (0.005)	0.858 (0.012)	0.856 (0.003)	0.857 (0.004)	0.940 (0.001)
ANN–RF–MWMOTE	0.850 (0.005)	0.867 (0.010)	0.852 (0.003)	0.858 (0.003)	0.940 (0.001)
ANN–RF–ADASYN	0.850 (0.008)	0.860 (0.015)	0.852 (0.006)	0.855 (0.004)	0.939 (0.003)

**Table 5:** Mean performance (and corresponding standard deviation) of phase I classifiers for 0.5-, 1-, 1.5-, 2-, 2.5-, and 3-year time-points. Top models are marked in **bold** for convenience.

Time-Point	Model	Sensitivity	Specificity	Accuracy	G-Mean	AUC
0.5-year	**GLM–LASSO–ADASYN**	**0.881 (0.001)**	**0.892 (0.001)**	**0.885 (0.000)**	**0.887 (0.000)**	**0.959 (0.000)**
	XGB–LASSO–ADASYN	0.876 (0.003)	0.883 (0.004)	0.879 (0.001)	0.880 (0.002)	0.958 (0.000)
	ANN–LASSO–ADASYN	0.885 (0.009)	0.883 (0.011)	0.884 (0.002)	0.884 (0.002)	0.965 (0.000)
1-year	GLM–LASSO–ADASYN	0.840 (0.001)	0.890 (0.002)	0.846 (0.000)	0.864 (0.001)	0.932 (0.000)
	XGB–LASSO–ADASYN	0.850 (0.002)	0.867 (0.004)	0.852 (0.001)	0.859 (0.002)	0.936 (0.000)
	**ANN–LASSO–ADASYN**	**0.851 (0.004)**	**0.888 (0.005)**	**0.856 (0.003)**	**0.870 (0.002)**	**0.943 (0.001)**
1.5-year	GLM–LASSO–ADASYN	0.859 (0.001)	0.857 (0.003)	0.859 (0.001)	0.858 (0.001)	0.933 (0.000)
	XGB–LASSO–ADASYN	0.867 (0.004)	0.855 (0.007)	0.867 (0.003)	0.861 (0.002)	0.940 (0.000)
	**ANN–LASSO–ADASYN**	**0.861 (0.005)**	**0.888 (0.011)**	**0.862 (0.004)**	**0.874 (0.003)**	**0.944 (0.001)**
2-year	GLM–LASSO–ADASYN	0.848 (0.001)	0.856 (0.003)	0.848 (0.001)	0.852 (0.001)	0.930 (0.000)
	XGB–LASSO–ADASYN	0.852 (0.003)	0.851 (0.006)	0.852 (0.003)	0.851 (0.002)	0.933 (0.000)
	**ANN–LASSO–ADASYN**	**0.856 (0.003)**	**0.863 (0.007)**	**0.857 (0.003)**	**0.860 (0.003)**	**0.940 (0.001)**
2.5-year	GLM–LASSO–ADASYN	0.838 (0.001)	0.829 (0.003)	0.838 (0.001)	0.833 (0.002)	0.919 (0.000)
	XGB–LASSO–ADASYN	0.850 (0.003)	0.821 (0.005)	0.850 (0.003)	0.835 (0.001)	0.922 (0.001)
	**ANN–LASSO–ADASYN**	**0.844 (0.009)**	**0.836 (0.017)**	**0.844 (0.009)**	**0.840 (0.005)**	**0.928 (0.002)**
3-year	GLM–LASSO–ADASYN	0.823 (0.001)	0.821 (0.003)	0.823 (0.001)	0.822 (0.001)	0.910 (0.000)
	XGB–LASSO–ADASYN	0.825 (0.004)	0.822 (0.011)	0.825 (0.004)	0.824 (0.005)	0.911 (0.001)
	**ANN–LASSO–ADASYN**	**0.839 (0.007)**	**0.819 (0.021)**	**0.839 (0.007)**	**0.829 (0.008)**	**0.918 (0.002)**

**Table 6:** Mean performance (and corresponding standard deviation) of survival month prediction (phase II) models that use LASSO or RF feature selection. Top models are marked in **bold** for convenience.

Model	RMSE	MAE
**GLM–LASSO**	**7.327 (0.001)**	**5.547 (0.007)**
GLM–RF	7.336 (0.001)	5.559 (0.007)
**XGB–LASSO**	**7.341 (0.007)**	**5.522 (0.010)**
XGB–RF	7.354 (0.008)	5.540 (0.009)
**ANN–LASSO**	**8.429 (1.552)**	**6.680 (1.741)**
ANN–RF	8.748 (1.617)	7.101 (1.937)

## Data Availability

The data used in this study can be requested from SEER: https://seer.cancer.gov/ (accessed on 22 July 2022).

## Supplementary Materials

In this study, the **R** programming language and **Tableau** were used to generate the results. In order to allow the researchers to reproduce our results, our codes and models are freely available online at: https://github.com/zahrame/LungCancerPrediction.

## Abbreviations

The following abbreviations are used in this manuscript:

SEER	Surveillance, Epidemiology, and End Results
GLM	General linear model
XGB	Extreme gradient boosting
ANN	Artificial neural network
SMOTE	Synthetic minority oversampling technique
RSLSMOTE	Relocating safe level SMOTE
BLSMOTE	Borderline SMOTE
ADASYN	Adaptive synthetic sampling
MWMOTE	Majority weighted minority oversampling technique
LASSO	Least absolute shrinkage and selection operator
RF	Random forest
WHO	World Health Organization
BDP	Bidirectional data partitioning
RNN	Recurrent neural network
CNN	Convolutional neural network
GBM	Gradient boosting machine
LR	Logistic regression
GLM	Generalized linear model
EV	Ensemble voting
MLP	Multilayer perceptron
BA	Bagging
DA	Dagging
ADB	AdaBoost
BOO	Boosting
RS	Random subspace
DS	Decision stump
SMO	Sequential minimal optimization
BN	Bayes Net
MBC	Model-based clustering
SOM	Self-ordering map
HC	Hierarchical clustering

NNMF	Non-negative matrix factorization
PCA	Principal component analysis
CE	Custom ensemble
Cox R	Cox regression
LB	LogitBoost
ADT	Alternating DT
ROC	Receiver operating characteristic
AUC	Area under the receiver operating characteristic curve
RMSE	Root mean squared error
MAE	Mean absolute error
OR	Odds ratio
